# Examining the Role of Sports Betting Marketing in Youth Problem Gambling

**DOI:** 10.1007/s10899-024-10347-x

**Published:** 2024-07-24

**Authors:** Gianluca Di Censo, Paul Delfabbro, Daniel L. King

**Affiliations:** 1 School of Psychology, The University of Adelaide, Adelaide, SA 5005 Australia; 2 College of Education, Psychology & Social Work, Flinders University, Adelaide, SA 5005 Australia

**Keywords:** Risk factors, Young people, Advertising, Inducements, In-play betting, Impulsivity

## Abstract

**Background**: Sports betting is becoming increasingly common among young people in the UK and Australia. There is a need to understand how the marketing of sports betting may influence risky and pathological gambling to inform policies aimed at reducing harm. This study examines whether sports betting advertising may predict problem gambling scores among young people, while accounting for non-marketing variables. **Methods**: We recruited 567 participants (53.1% male) aged 18–24 years from an online research panel. Participants were eligible if they had an active betting account and regularly bet on sports. We conducted a hierarchical regression analysis to examine whether four marketing-related measures (exposure to advertising, ad-driven betting decisions, use of betting inducements, and perceived susceptibility to betting inducements) could predict PGSI scores. We controlled for several demographic, psychological, and behavioural variables, including gender, gambling participation, spend per session, participation in in-play betting, normative beliefs about sports betting, and impulsivity. **Results**: The study revealed that sports betting marketing was positively associated with PGSI scores after controlling for non-marketing variables. Significant marketing predictors included ad-driven betting decisions and perceived influence from betting inducements. Other significant predictors included participation in non-sports betting gambling activities, spend per session, involvement in in-play betting, and the impulsivity trait of negative urgency. **Conclusion**: Sports betting marketing appears to be implicated in young people’s gambling problems. Specifically, young people who have gambling problems may be more likely to bet in response to advertising, and betting incentives may contribute to an intensification of their gambling behaviour. This study supports the implementation of regulations and restrictions on advertising as a measure to protect young problem gamblers.

## Introduction

Sports betting is an increasingly popular activity amongst young people. Research conducted in Australia has revealed that young people participate in sports betting at a higher rate than other age groups (Rockloff et al., 2020) and that it is one of the most popular forms of gambling among this demographic, with only casino table games surpassing its popularity (Armstrong & Carroll, 2017). Large-scale surveys have consistently shown that younger individuals are more susceptible to experiencing gambling harm compared to other age groups (Armstrong & Carroll, 2017; Australian Gambling Research Centre, 2023; Gambling Commission, 2022; Rockloff et al., 2020). For example, Armstrong and Carroll (2017) found that although young people represent only 22.5% of the total population, they account for 34.3% of problem sports bettors. In the same report, it was found that 89.7% of problem gamblers who identified themselves as sports bettors were male. Together, these findings indicate that younger males face a greater risk of experiencing gambling-related issues, specifically in relation to sports betting (Hing, Russell, & Browne, 2017). This has been attributed to psychological factors, which differ by gender and age (Delfabbro & King, 2012), but also broader socio-ecological factors, including the high level of marketing associated with sports betting activities. The proliferation of wagering advertising has led countries such as Belgium, Italy, and Spain to implement stricter advertising regulations to mitigate sports betting harm, particularly for young people (*Divieto di pubblicità di giochi con vincite in denaro online*, 2018; Strauss, 2023; Yogonet, 2021), and other countries, such as Australia, are considering similar restrictions (Parliament of Australia, 2023). To strengthen the evidence supporting these measures, it is pertinent that researchers investigate whether sports betting marketing contributes to problem gambling among young sports bettors.

### Demographic and Psychological Risk Factors

A number of studies have found that young people are more prone to encountering issues related to gambling (Armstrong & Carroll, 2017; Delfabbro & King, 2012; Hing et al., 2015, 2016), and specifically related to sports betting (Hing, Russell, & Browne, 2017). Furthermore, multiple studies have found an association between being male and experiencing gambling-related problems (Hing, Russell, & Browne, 2017; Hing et al., 2016 ; Russell, Hing, Li et al., 2018). One explanation for this is that younger individuals, particularly younger men, generally exhibit a higher propensity for engaging in risky behaviours overall, such as binge drinking and illicit drug use (Australian Institute of Health and Welfare, 2020). For example, the trait of impulsivity is commonly linked to various negative consequences, including addiction, eating disorders, and other mental health disorders (Berg et al., 2015; Dir et al., 2013; Mitchell & Potenza, 2014), and this extends to gambling (Hing, Li, et al., 2017; Russell, Hing, Li et al., 2018). Specific components of impulsivity, namely negative and positive urgency, have exhibited a strong correlation with the occurrence of problem gambling. Positive urgency, which refers to the tendency for people to make rash decisions when they are in a positive emotional state (Cyders & Smith, 2007), has been found to be associated with problem gambling (Howe et al., 2019). Negative urgency, in contrast, refers to the tendency for people to act impulsively when experiencing negative emotions (Cyders & Smith, 2007), and several studies have shown that it is associated with problem gambling (Blain et al., 2015; Haw, 2015; MacLaren et al., 2011; Savvidou et al., 2017). Negative urgency is a significant factor in gambling behaviour as it is often motivated by the desire to alleviate negative emotions, and research has shown that experiencing negative mood states increases the likelihood of being a high-risk gambler (Hing, Russell, & Browne, 2017; Price, 2020).

### Behavioural Risk Factors

Several betting-related behavioural risk factors have been found to be associated with gambling problems. The frequency of participation in sports betting has been found to be a robust risk factor for gambling problems among sports bettors (Hing, Russell, & Browne, 2017; Hing et al., 2016; Lopez-Gonzalez, Estévez et al., 2018; Russell, Hing, Li et al., 2018). In addition, Hing, Russell and Browne (2017) found that sports bettors who participated in a variety of different betting activities were significantly more likely to be classified as problem gamblers. Previous research has indicated that sports and race bettors are more likely to be classified as problem gamblers when they have a larger betting expenditure (Hing et al., 2019a; Russell, Hing, Li et al., 2018). Furthermore, studies have suggested that in-play sports betting is a particularly problematic form of betting and have shown evidence that it is associated with problem gambling (e.g., Hing et al., 2018; Lopez-Gonzalez, Estévez et al., 2018; Lopez-Gonzalez, Griffiths et al., 2018; Vieira et al., 2023). This is likely because it offers higher event frequency and a more continuous betting experience, with shorter intervals between placing a bet and receiving an outcome than traditional sports betting (Parke & Parke, 2019). Additionally, online betting platforms facilitate access to sports betting across all hours of the day, giving bettors access to an array of betting opportunities that comprise a variety of sports as well as exotic and complex betting types.

### Sports Betting Marketing

Advertising for sports betting is pervasive during sports betting broadcasts, through both direct marketing and sponsorships (Bunn et al., 2018; Deans et al., 2017; Djohari et al., 2019; Newall et al., 2019; Thomas et al., 2016). However, advertising also appears on internet advertising banners, social media feeds, and through individualised marketing avenues such as push notifications and text messages. Additionally, more subtle forms of advertising exist such as affiliate marketing and content advertising, which have not been well researched. Therefore, there are a plethora of advertising channels through which young people may be exposed to sports betting. The pervasiveness of sports betting advertising and its integration into sports have been identified as contributing to the perception of its social acceptability, especially among men (Deans et al., 2017; Lopez-Gonzalez et al., 2017; Marko et al., 2022). Additionally, there are suggestions that frequent reminders about betting may have a negative impact on individuals with gambling problems, potentially encouraging them to continue their gambling behaviour or impeding their efforts to stop (Hing et al., 2014; Lopez-Gonzalez et al., 2019; Parke & Parke, 2019). This is because advertising may potentially trigger gambling urges in individuals who are strongly conditioned to gamble (Binde, 2008). This is not dissimilar to how drug, alcohol, and nicotine users develop strong cue-elicited cravings when they encounter stimuli associated with their preferred substance (Childress et al., 1993; Vafaie & Kober, 2022).

Bookmakers employ promotional strategies to incentivise betting, which are commonly referred to as betting inducements. These inducements have been shown to intensify betting and are more commonly used by higher-risk sports bettors (Deans et al., 2017; Hing et al., 2014; Hing, Russell, Lamont et al., 2017; Hing et al., 2018; Lopez-Gonzalez et al., 2019). Inducements can be effective through a number of mechanisms. For instance, incentives such as free bets or bet credits are often given to bettors as a reward for their loyalty, signing up with the operator, or making a deposit. These incentives are designed to offer bettors additional opportunities to place bets and extend their play, potentially further reinforcing their betting behaviours (Hing et al., 2014). Free bets and bet credits are often perceived as more advantageous than they truly are (Deans et al., 2017; Killick & Griffiths, 2020). Typically, bettors only receive the winnings from these bets, and there may be multiple play-through requirements that must be met before funds can be withdrawn. The early payout offer, another type of betting inducement, provides bettors with the option to withdraw their winnings sooner (i.e., before the end of the event). This feature is primarily beneficial to those who intend to re-stake their earnings immediately, such as in-play bettors. From the perspective of the betting companies, incentives are likely used as a tool to further differentiate themselves from competitors. However, it is likely that betting operators use inducements to remind customers to sustain their betting habits by regularly offering them largely superficial incentives.

### The Current Study

The current evidence indicates that advertising for sports betting can influence people’s participation in sports betting and contribute to gambling-related harm. Since sports betting marketing is considered to be the most preventable of all the risk factors discussed, many academics have supported a ban or stricter regulations (e.g., Deans et al., 2017; Hing et al., 2019b; Lopez-Gonzalez et al., 2019; Russell, Hing, Browne, et al., 2018). However, a limited number of studies have examined its influence specifically on young people, and there are additional variables that are closely linked to problem gambling and may potentially serve as more robust predictors. Therefore, it still needs to be determined whether advertising for sports betting contributes to youth problem gambling when considering the influence of other known risk factors. In the current study, we sought to examine the potential for sports betting marketing to predict problem gambling scores among young sports bettors. We considered several demographic, psychological, and behavioural risk factors in our analysis as comparator variables. We examined whether weekly exposure to betting ads, ad-driven bet decisions, the use of inducements, and susceptibility to betting inducements are associated with problem gambling scores. Based on past research, it was hypothesised that betting marketing would be positively associated with problem gambling scores after controlling for other, non-marketing-related factors.

## Method

### Participants

Participants were recruited from the online research panel, Prolific. The eligibility criteria required that individuals be aged between 18 and 24, have participated in sports betting within the past 12 months, and possess an active betting account. A total of 649 participants agreed to take part in the study. The study was exclusively promoted to individuals between 18 and 24 on the Prolific platform. We allowed participants to self-select whether they met the remaining inclusion criteria (i.e., bet on sports in the past 12 months and hold an active betting account). However, those who did not meet the criteria (*n* = 58) were excluded from the data analysis (detailed in the Analytic Strategy below). Table 1 provides a summary of participants’ demographic information.

### Sample Size

The sample size for our study was determined using a rule of thumb for regression analysis, which suggests a minimum of 10 observations per predictor variable (Wilson Van Voorhis & Morgan, 2007). To account for the 33 variables that could serve as predictors in our model, we aimed to obtain a sample size of at least 330 participants. We recruited 649 participants, anticipating excluding those who did not fully satisfy the inclusion criteria or had poor-quality responses.

### Measures

In addition to gathering basic demographic information (provided in Table 1), the following standard measures were used:

#### Problem Gambling Score

We used the *Problem Gambling Severity Index* (PGSI) to determine participants’ problem gambling scores (Ferris & Wynne, 2001). The scale consists of nine items and provides four response options, ranging from never (0) to almost always (3). It has a score range of 0–27 and classifies individuals into four categories: non-problem gamblers (score of 0), low-risk gamblers (score of 1–2), moderate-risk gamblers (score of 3–7), and problem gamblers (score of 8 or higher). In our sample, the measure showed a high level of internal consistency, with a Cronbach’s alpha of 0.89.

#### Impulsivity

We evaluated participants’ impulsivity using the *Short UPPS-P Impulsive Behaviour Scale* (SUPPS-P) (Cyders et al., 2014). The SUPPS-P consists of five sub-scales, each pertaining to different aspects of impulsivity: *positive urgency*, *negative urgency*, *lack of premeditation*, *lack of perseverance*, and *sensation seeking*. The scale consists of 20 statements, to which participants indicate their level of agreement using a 4-point scale ranging from strongly agree (0) to strongly disagree (3). It has a score range of 0–60 for the total measure, while each sub-scale has a range of 0–12. Cronbach’s alpha coefficients were: 0.80 for *positive urgency*, 0.60 for *sensation seeking*, 0.81 for *lack of premeditation*, 0.71 for *lack of perseverance*, and 0.79 for *negative urgency*.

#### 12-Month Frequency of Sports Betting Participation

Our intention was to collect data from participants regarding their frequency of engagement in sports betting while minimising recall bias. Given the varying frequencies at which our participants typically engaged in sports betting, we allowed them to choose a reporting period that best corresponded to their typical betting patterns. The reporting periods cover betting for the previous week, month, or 12 months. To obtain a standardised 12-month frequency of participation, we multiplied the reported betting frequencies of participants who provided data for the past week by 52 and for the past month by 12.

#### Participation in Non-Sports Betting Gambling Activities

We employed the *Gambling Behaviours (GB) Scale* to assess the frequency with which participants engaged in non-sports betting gambling activities. This eight-item scale assesses participation in various forms of gambling, such as Electronic Gaming Machines (EGMs), card games, and the lottery, among others. While sports betting was included in the scale presented to the participants, it was removed from the analysis to focus exclusively on participation in other forms of gambling. Participants indicated their responses using a 7-point frequency scale, which allowed for score ranges of 0–48. The scales’ Cronbach’s alpha was 0.75.

#### Weekly Sports Betting Advertising Exposure

The *Sports Betting Advertising Exposure Scale* was used to measure the amount of sports betting advertising that participants were exposed to in the past week. This measure assesses exposure through multiple channels, including *Television*, *Radio*, *Print*, *Outdoor advertising*, *Social media feeds*, *Email or text message advertising*, *Push notifications*, *Internet advertising banners*, *Podcasts*, and *During sports commentary*. Participants were asked to indicate the number of ads they encountered in the past week using a sliding scale ranging from 0 to 25. Total scores could range from 0 to 250. The scale had a Cronbach’s alpha of 0.83 in the current sample, indicating a high level of internal consistency.

#### Ad-Driven Betting Decisions

We measured participants’ perceived likelihood of making a decision to bet based on sports betting advertising using the *Ad-Driven Betting Decision Scale*. Participants rated their level of agreement with four statements using a 7-point Likert scale. The scores on the scale can range from 0 to 24. The scale had a Cronbach’s alpha of 0.91 in the current sample, indicating a high level of internal consistency among items.

#### Inducements Used in the Past 3 Months

Participants indicated how often they used various betting inducements in the past 3 months using a text box. The inducements included: sign-up offers, deposit matches, bonus bets, increased odds offers, reduced risk/stake back offers, extra chance opportunities, and cash-out opportunities. For inferential testing, these variables were converted into binary variables with two levels: *Used* and *Not used*.

#### Perceived Susceptibility to Betting Inducements

We used the 10-item *Inducement Susceptibility Scale* to assess the perceived impact of betting inducements on participants’ higher-risk betting behaviours, such as loss chasing, placing riskier bets, and self-control. Participants indicated their level of agreement using a 7-point Likert scale. Scores on the scale can range from 0 to 60. The scale’s Cronbach’s alpha was 0.94, indicating strong internal consistency.

#### Sports Betting Normative Beliefs

Participants’ beliefs about the social norms of sports betting were captured through the 10-item *Sports Betting Normative Beliefs Scale*. This measure assessed young people’s perceptions of both injunctive and descriptive social norms associated with sports betting as well as other social normative influences such as social desirability, social identity, and societal values. It considered key reference groups, including society at large, the individual’s peers, and their friends and family. Participants indicated their responses using a 7-point Likert scale. The scale allowed for potential scores to range from 0 to 60. Three items were negatively phrased and thus were reverse scored. The Cronbach’s alpha coefficient in the current sample was 0.69 which is a borderline level of internal consistency.

#### Number of Sports Betting Accounts

Participants were asked about the number of active betting accounts they held and provided their answers in a text box.

#### Participation in In-Play Betting

Typical participation in in-play or *live* betting was evaluated using a single-item question that offered response options of: *Never*, *Rarely*, *Sometimes*, and *Often*. For inferential testing, the responses were dichotomised into *Never* and *Yes*.

#### Typical Spend Per Session

The data on typical spend was collected using a text box in which participants reported how much they spent during a typical betting session. It was emphasised that the figure should represent the amount of money wagered, excluding any gains or losses resulting from the outcomes of bets.

#### Other Measures

In addition to the measures mentioned, the participants also completed the ad hoc measure *Sports Betting Advertising Normative Influence Scale* and a measure capturing their estimation of the prevalence of youth engagement in sports betting. However, the results of these measures were not reported in the current study.

### Procedure

The survey was hosted on Qualtrics. The study involved participants reading an information sheet about the study, after which they provided their informed consent. Following that, they proceeded to complete the measures. Each participant received a reimbursement of £1.50 as compensation for their participation. On average, it took participants 8 min and 42 s to complete the survey. The current study was approved by the Human Research Ethics Subcommittee of the University of Adelaide’s School of Psychology; project number: H-2023/88.

### Analytic Strategy

We screened the data to determine whether all participants met the eligibility criteria. Participants had to confirm that they had bet on sports at least once in the past year on two measures: (1) Using an open-ended text box, participants reported their sports betting frequency in the previous year; and (2) the GB Scale included a question asking participants to estimate their sports betting frequency. In addition, participants were asked to disclose the number of active betting accounts they held to determine their eligibility under the second inclusion criterion (i.e., having an active betting account). A total of 82 participants were identified and excluded for not meeting these criteria. We used box plots and z-score transformations to identify outliers in the data. We addressed the non-normal distribution of the dependent variable, the PGSI, by employing a logarithmic (+ 1) transformation, which successfully normalised its distribution.

The primary research question sought to determine whether sports betting marketing would predict PGSI scores when accounting for the influence of established behavioural, psychological, and demographic predictors. To test this, a hierarchical regression analysis was conducted. The first step involved entering established predictors, and the second step involved including a set of sports betting marketing predictors. If the second step explained further variance in PGSI scores compared to the first step, it could be determined that marketing has an influence on PGSI. The secondary research question was aimed at identifying the specific marketing predictors that had the greatest impact on PGSI scores. We analysed this research question by determining which predictors, added in the second step of the model, significantly contributed to the model and had the highest beta coefficients. The results section provides specific details on how we assessed the model assumptions for hierarchical regression.

## Results

### Descriptive Statistics

The study comprised 567 participants aged 18 to 24. The sample consisted of slightly more males (53.1%) than females. Participants most often held an undergraduate degree (40.7%), were employed (60.3%), lived with their parents (52.2%), and were unmarried (95.6%). Most participants were born in the UK (88.4%) and reported English as their primary language spoken at home (95.8%). The two largest groups in the sample, based on problem gambling score categories, were low-risk gamblers (35.3%) and non-problem gamblers (30.0%). A smaller, yet notable, portion of the sample included moderate-risk gamblers (22.8%) and problem gamblers (12.0%). Although 39.0% did not participate, a significant portion of the participants engaged in in-play betting to different extents. For detailed data, refer to Table 1.

**Table 1:** Basic descriptive statistics of the sample

	*n*	%
Gender		
Male	301	53.1
Female	258	45.5
Non-binary/third gender	7	1.2
Prefer not to say	1	0.2
Problem Gambling Score Category		
Non-problem	170	30.0
Low-risk	200	35.3
Moderate-risk	129	22.8
Problem gambler	68	12.0
Education		
Secondary or less	93	16.4
Vocational or some university	174	30.7
Undergraduate degree	231	40.7
Postgraduate degree	69	12.2
Work status		
Employed	342	60.3
Unemployed	63	11.1
Student	162	28.6
Living circumstance		
With Parents	296	52.2
Independently	271	47.8
Marital status		
Not married	542	95.6
Married	25	4.4
Born in the UK		
No	66	11.6
Yes	501	88.4
Speak English at home		
No	24	4.2
Yes	543	95.8
Participate in live or in-play betting		
Never	221	39.0
Rarely	183	32.3
Sometimes	121	21.3
Often	42	7.4
Total	567	100

Table 2 presents a summary of variables related to gambling, grouped by the PGSI categories as well as for the total sample. Given the variability of the data, means and medians are reported along with standard deviations (*SD*) and interquartile ranges (*IQR*). On average, participants reported betting on sports 12 times in the past year, typically spending £10 per betting session, having two betting accounts, and having used four betting inducements in the past three months. Problem gamblers participated in sports betting more frequently (*Mdn* = 48, *IQR* = 81) when compared to other gamblers. They also spent more per betting session (*Mdn* = £20, *IQR* = 15), had the most betting accounts (*Mdn* = 3, *IQR* = 4.00), and used the most sports betting inducements (*Mdn* = 12, *IQR* = 14). Problem gamblers, in addition, reported believing that sports betting was more normative (*M* = 28.81, *SD* = 8.44), encountering more sports betting advertising per week (*M* = 45.40, *SD* = 35.74), perceiving that they were more susceptible to making betting decisions driven by advertising (*M* = 12.74, *SD* = 7.13), and perceiving sports betting inducements influenced their behaviours more (*M* = 36.95, *SD* = 12.61). Non-problem gamblers had the lowest means and medians for all variables.

**Table 2:** Descriptive statistics for gambling-related variables

	M	SD	Mdn	IQR
12-Month Sports Betting Frequency				
Non-problem	17.58	28.74	5.00	22.00
Low-risk	27.38	34.65	12.00	32.00
Moderate-risk	33.75	39.77	22.00	44.00
Problem gambler	63.21	49.94	48.00	81.00
Total	28.59	37.54	12.00	35.00
Participation in Other Gambling Activities				
Non-problem	5.84	5.03	5.00	6.00
Low-risk	6.61	4.96	6.00	6.00
Moderate-risk	9.89	5.92	8.50	7.00
Problem gambler	15.88	9.26	15.50	13.50
Total	7.85	6.35	7.00	7.00
Typical Spend Per Betting Session (£)				
Non-problem	10.27	10.43	5.00	5.00
Low-risk	14.78	13.20	10.00	15.00
Moderate-risk	17.38	14.05	10.00	14.38
Problem gambler	23.98	14.68	20.00	15.00
Total	14.64	13.25	10.00	15.00
Number of Sports Betting Accounts				
Non-problem	2.42	3.11	1.00	1.00
Low-risk	3.07	3.46	2.00	2.00
Moderate-risk	3.25	3.27	2.00	2.00
Problem gambler	3.90	2.92	3.00	4.00
Total	2.97	3.28	2.00	2.00
Sports Betting Normative Beliefs				
Non-problem	22.40	7.36	24.00	11.00
Low-risk	23.72	7.07	23.50	9.25
Moderate-risk	25.45	6.70	25.00	8.00
Problem gambler	26.81	8.44	28.50	11.50
Total	23.92	7.33	24.00	10.00
Weekly Sports Betting Advertising Exposure				
Non-problem	27.25	24.37	20.00	25.50
Low-risk	31.81	24.11	26.00	28.00
Moderate-risk	40.53	29.10	35.00	32.00
Problem gambler	45.40	35.74	34.00	44.25
Total	33.33	27.08	26.00	32.00
Ad-Driven Betting Decisions				
Non-problem	4.82	4.54	4.00	8.00
Low-risk	8.41	5.37	8.00	9.00
Moderate-risk	10.10	5.71	10.50	9.75
Problem gambler	12.74	7.13	15.50	10.50
Total	7.96	5.91	7.00	9.00
Number of Inducements Used				
Non-problem	3.67	6.24	2.00	4.00
Low-risk	6.59	9.28	4.00	7.00
Moderate-risk	10.72	12.18	7.00	12.00
Problem gambler	15.26	15.87	12.00	14.00
Total	7.25	10.47	4.00	9.00
Susceptibility to Sports Betting Inducements				
Non-problem	11.19	10.85	10.00	16.00
Low-risk	21.41	12.31	22.00	19.00
Moderate-risk	29.52	11.39	30.00	16.00
Problem gambler	36.95	12.61	38.00	16.50
Total	21.10	14.31	21.00	23.00

### Inferential Statistics

#### Bivariate Correlations

Table 3 displays the bivariate correlations between logarithmic transformed PGSI scores and a number of variables. Several gambling behavioural measures, such as *12-month sports betting frequency* (*r* = .28, *p* < .01), *participation in other gambling activities* (*r* = .43, *p* < .01), *typical spend per betting session* (*r* = .29, *p* < .01), and *in-play betting* (*r* = .36, *p* < .01) were found to have significantly positive correlations with the PGSI. Among sports betting advertising variables, *susceptibility to sports betting inducements* showed the strongest positive correlation with the PGSI (*r* = .63, *p* < .01). Additionally, *weekly sports betting advertising exposure* (*r* = .26, *p* < .01) and *ad-driven betting decisions* (*r* = .46, *p* < .01) each showed strong associations with the PGSI. The use of each sports betting inducement was positively associated with the PGSI score. The study revealed significant positive correlations between the different aspects of impulsivity, specifically *negative urgency* (*r* = .33, *p* < .01), *lack of premeditation* (*r* = .27, *p* < .01), and *positive urgency* (*r* = .32, *p* < .01). In relation to demographic factors, the male gender was associated with the PGSI score (*r* = .16, *p* < .01), while other variables such as level of education, employment status, and living circumstance did not show significant correlations with the PGSI (Table 3).

**Table 3:** Bivariate correlations of proposed predictors with PGSI score

	PGSI Score	
12-Month Sports Betting Frequency		0.28^**^
Participation in Other Gambling Activities		0.43^**^
Typical Spend Per Betting Session		0.29^**^
Participation in ‘In-Play’ Betting (ref. Never)		0.36^**^
Number of Sports Betting Accounts		0.12^**^
Gender (ref. Female)		0.16^**^
Vocational or Some University (ref. Secondary or Less)		0.05
Undergraduate Degree (ref. Secondary or Less)		0.06
Postgraduate Degree (ref. Secondary or Less)		0.00
Unemployed (ref. Employed)		− 0.01
Student (ref. Employed)		0.03
Living Circumstance (ref. With parents)		0.03
Marital (ref. Not married)		0.07
Weekly Income		0.05
Born in the UK (ref. No)		− 0.03
English Spoken at Home		− 0.04
Negative Urgency		0.34^**^
Lack of Perseverance		0.04
Lack of Premeditation		0.27^**^
Sensation Seeking		0.09^*^
Positive Urgency		0.32^**^
Sports Betting Normative Beliefs		0.26^**^
Weekly Sports Betting Advertising Exposure		0.26^**^
Ad-Driven Betting Decisions		0.46^**^
Sign Up Used		0.18^**^
Deposit Match Used		0.25^**^
Bonus Bet Used		0.32^**^
Increased Odds Used		0.32^**^
Reduced Risk Used		0.25^**^
Extra Chance Used		0.27^**^
Cash Out Used		0.28^**^
Susceptibility to Sports Betting Inducements		0.62^**^

Note. ** *p* < .01 (2-tailed), * *p* < .05 (2-tailed)

#### Hierarchical Regression Model

We conducted a hierarchical regression analysis using the PGSI as the outcome variable and the significant bivariate correlates of the PGSI (see Table 3) as the predictor variables. The analysis consisted of two stages. In the first stage, non-marketing-related predictors of the PGSI were introduced, and in the second stage, marketing-related variables were added (see Table 4). *Sensation seeking* was excluded from the regression model due to its weak correlation with the PGSI and to maintain model parsimony. A sensitivity analysis further confirmed that the model’s fit improved without this variable. The model did not show any signs of multicollinearity, as indicated by the VIF and tolerance statistics.

In the first step of the model, the predictors explained 34.4% of the variance (*R*² = 0.344, *F*(10, 456) = 23.862, *p* < .001). The adjusted *R²* was 0.329, and the standard error of the estimate was 0.273. The variables that made a significant contribution to the model were *12-month sports betting frequency*, *frequency of engaging in non-sports betting gambling activities*, *typical spend*, *participation in in-play betting*, and the psychological trait of *negative urgency*. In the second step of the model, an additional 15.0% of the variance was explained (Δ*R²* = 0.150, *F* change (10, 446) = 13.227, *p* < .001). The total variance explained by the second step was 49.4%, with an adjusted *R²* of 0.471 and a standard error of the estimate of 0.242. These findings indicate that there was a relationship between sports betting marketing-related variables and PGSI when controlling for the influence of psychological, behavioural, and demographic variables.

The variables related to marketing that significantly contributed to the model were the two psychological variables: *ad-driven betting decisions* and *perceived susceptibility to betting inducements*. Both variables were positively associated with the PGSI, which signifies that, while holding other variables constant, those who perceive that they make more ad-driven betting decisions and perceive themselves as being more susceptible to inducements were more likely to have higher scores on the PGSI. Notably, *normative beliefs about sports betting*, despite not being a significant predictor of PGSI in the first step of the regression, emerged as a significant predictor in the second step. The negative beta coefficients indicate that an increase in the belief that sports betting is normal is associated with a decrease in PGSI scores, holding all other variables constant. Among the variables that significantly contributed to the first step of the model, only *12-month sports betting frequency* failed to do so in the second step. See Table 4 for detailed statistics.

**Table 4:** Hierarchical Regression Analysis: Predicting PGSI using Non-Marketing Predictors (First Step) and Marketing Predictors (Second Step)

Variable	*B*	*SE*	β	*t*	*p*	95% *CI*
Step 1						
(Intercept)	-0.42	0.07		-5.81	< 0.001	[-0.57, − 0.28]
12-Month Sports Betting Frequency	0.00	0.00	0.10	2.13	0.033	[0.00, 0.00]
Participation in Other Gambling Activities	0.01	0.00	0.21	4.85	< 0.001	[0.01, 0.02]
Typical Spend	0.01	0.00	0.19	4.84	< 0.001	[0.00, 0.01]
Participation in ‘In-Play’ Betting (ref. Never)	0.15	0.03	0.23	5.54	< 0.001	[0.10, 0.21]
Number of Sports Betting Accounts	0.00	0.01	-0.02	-0.43	0.665	[-0.02, 0.01]
Gender (ref. Female)	0.04	0.03	0.06	1.37	0.171	[-0.02, 0.09]
Negative Urgency	0.02	0.01	0.19	3.55	< 0.001	[0.01, 0.03]
Lack of Premeditation	0.01	0.01	0.04	1.01	0.313	[-0.01, 0.02]
Positive Urgency	0.01	0.01	0.09	1.69	0.091	[-0.00, 0.02]
Sports Betting Normative Beliefs Scale	0.00	0.00	0.00	0.01	0.996	[0.00, 0.00]
Step 2						
(Intercept)	-0.21	0.07		-3.01	0.003	[-0.34, − 0.07]
12-Month Sports Betting Frequency	0.00	0.00	0.05	1.15	0.25	[0.00, 0.00]
Participation in Other Gambling Activities	0.01	0.00	0.14	3.45	0.001	[0.00, 0.01]
Typical Spend	0.00	0.00	0.13	3.55	< 0.001	[0.00, 0.01]
Participation in ‘In-Play’ Betting (ref. Never)	0.09	0.03	0.14	3.62	< 0.001	[0.04, 0.15]
Number of Sports Betting Accounts	0.00	0.01	-0.03	-0.74	0.462	[-0.02, 0.01]
Gender (ref. Female)	0.04	0.03	0.05	1.41	0.16	[-0.01, 0.08]
Negative Urgency	0.02	0.01	0.14	3.02	0.003	[0.01, 0.03]
Lack of Premeditation	0.00	0.01	0.02	0.61	0.543	[-0.01, 0.02]
Positive Urgency	0.00	0.01	0.01	0.11	0.913	[-0.01, 0.01]
Sports Betting Normative Beliefs Scale	-0.01	0.00	-0.14	-3.63	< 0.001	[-0.01, 0.00]
Weekly Sports Betting Advertising Exposure	0.00	0.00	0.00	0.08	0.938	[0.00, 0.00]
Ad-Driven Betting Decisions	0.01	0.00	0.10	2.08	0.038	[0.00, 0.01]
Sign Up Used	-0.05	0.03	-0.08	-1.91	0.057	[-0.10, 0.00]
Deposit Match Used	-0.01	0.03	-0.01	-0.37	0.71	[-0.07, 0.05]
Bonus Bet Used	0.03	0.03	0.05	1.10	0.273	[-0.03, 0.09]
Increased Odds Used	0.02	0.03	0.03	0.79	0.430	[-0.03, 0.08]
Reduced Risk Used	0.00	0.03	0.01	0.14	0.891	[-0.06, 0.07]
Extra Chance Used	0.03	0.04	0.03	0.86	0.392	[-0.04, 0.11]
Cash Out Used	0.02	0.03	0.03	0.77	0.444	[-0.03, 0.07]
Susceptibility to Sports Betting Inducements	0.01	0.00	0.41	7.86	< 0.001	[0.01, 0.01]

Note. For Step 1, *R²* = 0.344, Adjusted *R²* = 0.329, *F*(10, 456) = 23.862, *p* < .001, *SE* = 0.273. For Step 2, *R²* = 0.494, Adjusted *R²* = 0.471, *ΔR²* = 0.150, *F* change (10, 446) = 13.227, *p* < .001, *SE* = 0.242. Durbin-Watson = 2.084. *B* = unstandardised regression coefficient, *SE* = standard error, β = standardised regression coefficient, *t* = *t* value, *p* = *p* value, *CI* = Confidence Interval

## Discussion

The current study sought to examine the psychological, behavioural, demographic, and marketing-related factors that might contribute to problem gambling among young people who regularly engage in sports betting. We observed that engagement in sports betting and other forms of gambling, as well as the amount typically spent per session, participation in in-play betting, and the psychological trait negative urgency were all related to PGSI scores. The ad-driven betting decisions and susceptibility to inducements variables were found to be significant predictors of PGSI scores, while the quantifiable measures of advertisement exposure and use of inducements were not. This suggests that young people who believe they are influenced by sports betting marketing are more likely to experience gambling problems, regardless of how much they are exposed to or engaged with it. The analysis demonstrated that susceptibility to sports betting inducements was the strongest predictor of PGSI scores. This suggests that those who believe that sports betting inducements influence them to engage in riskier gambling behaviours are particularly likely to have higher problem gambling scores. Overall, this study found that sports betting marketing may contribute to problem gambling.

### Behavioural and Psychological Factors

Previous studies have found that those who are highly engaged in sports betting are more likely to experience gambling-related problems than non-participants (Hing et al., 2016; Hing, Russell, & Browne, 2017; Lopez-Gonzalez, Estévez et al., 2018). We observed that greater engagement with sports betting was a significant predictor of problem gambling. However, when controlling for marketing-related variables, it was no longer a significant predictor. A possible explanation is that individuals who perceived themselves to be more susceptible to betting inducements were both likely to bet more frequently and experience greater gambling-related harm. This indicates that susceptibility to betting inducements could account for some of the variance in PGSI scores that was previously attributed to frequency of betting participation while also explaining its own unique variance in PGSI related to engagement in riskier betting behaviours. However, engaging in non-sports betting gambling activities remained a significant predictor in the later stages of the analysis, which suggests that young people who engage in other gambling activities are more likely to experience gambling problems. This supports previous research indicating that engaging in multiple forms of gambling is strongly associated with an increased likelihood of experiencing gambling-related problems (Dowling et al., 2017; Hing et al., 2016).

Existing research has consistently shown that problem gambling is associated with impulsivity (e.g., Hing, Li, et al., 2017; Howe et al., 2019; Russell, Hing, Li et al., 2018). Negative urgency, a subtype of impulsivity, has consistently shown an association with addictive behaviours and psychopathologies (Berg et al., 2015; Dir et al., 2013; Mitchell & Potenza, 2014). The current study found that negative urgency is a significant risk factor for problem gambling, which corroborates the findings of past research (Blain et al., 2015; Haw, 2015; MacLaren et al., 2011; Savvidou et al., 2017). This suggests that problem gamblers may be motivated to gamble during negative moods, even in the face of negative consequences. Sports betting activities are increasingly able to include features that can encourage impulsive betting. These include frequent and targeted push and SMS notifications, in-play betting, complex combination betting across different activities and the capacity to offer products across much of the day.

In-play betting appears to be a particular activity of interest and was found to be related to problem gambling scores, a finding that has also been obtained in multiple other studies (e.g., Hing et al., 2018; Lopez-Gonzalez, Estévez et al., 2018; Lopez-Gonzalez, Griffiths et al., 2018; Vieira et al., 2023). The defining feature of in-play betting is the considerably shorter interval between placing bets and discovering the outcome, especially when compared to traditional sports betting. Moreover, in-play betting frequently taps into the numerous microevents during sports matches, thereby increasing the bettors’ wagering opportunities (Russell, Hing, Browne, et al., 2018). In-play betting offers bettors the ability to place a wager, obtain a return, and immediately re-stake the return in another wager, leading to a potential cycle of continuous betting (Parke & Parke, 2019). Essentially, this gives bettors an experience resembling the continuous and consistent positive reinforcement observed in EGMs. Additionally, the continual and concurrent betting opportunities posed by in-play betting demand greater cognitive engagement from bettors, further immersing them in the betting experience. In essence, individuals with gambling problems tend to exhibit impulsive behaviours when experiencing negative emotions, and we propose that in-play betting facilitates this tendency by providing a means for escapism and distraction from negative moods, owing to its consistent and continuous reinforcement.

Interestingly, in the second step of the regression model, *sports betting normative beliefs* became a significant predictor, despite not being significant in the first step. The relationship between sports betting normative beliefs and the PGSI was negative. Thus, when controlling for the influence of marketing-related variables, individuals with higher PGSI scores tended to view sports betting as less socially normal and acceptable. This is noteworthy considering that in the bivariate correlation analysis, sports betting normative beliefs had a positive relationship with PGSI scores, and in numerous other studies, normative influences were associated with problem gambling (e.g., Hing et al., 2016; Howe et al., 2019; Russell, Hing, Li et al., 2018). Consequently, based on this finding, it may be that individuals with higher PGSI scores both perceive themselves as being more susceptible to sports betting marketing and are less likely to view sports betting as a normal or socially acceptable activity. This may be due to the tendency for highly involved sports bettors, or those with gambling problems, to associate with other people, or “cliques” who are similarly highly involved (e.g., by participating in online betting-related forums) and, thus, perceive sports betting as atypical or uncommon.

### Sports Betting Marketing

In our study, the amount of advertising exposure was not a predictor of gambling problems. While more engaged gamblers might encounter increased sports betting advertising, exposure does not seem to play a significant role in their gambling issues. One possible explanation is that, in established markets, advertising efforts primarily focus on increasing the company’s market share by attracting customers from competitors rather than significantly changing overall consumption or intensifying behaviour. This is referred to as *selective demand* or *competitive* advertising (Bass et al., 2005; Schultz & Wittink, 2018). However, in nascent markets, the role of advertising is to generate greater demand for the product, which is known as *primary demand* or *generic* advertising (Bass et al., 2005; Schultz & Wittink, 2018). Therefore, we may observe that advertising increases the betting behaviour of those new to the betting market, while, for experienced bettors, advertising may function to influence the bookmaker with whom they choose to bet.

Additionally, people who attempt to reduce their betting may begin to experience an increase in targeted advertising (e.g., through push notifications and text messages) in an effort for betting companies to re-engage them in betting. Thus, when considering other variables, the amount of advertising encountered may not always be associated with the frequency of engagement or problem gambling scores. It is conceivable, nevertheless, that betting inducements may potentially intensify gambling behaviour by making betting more affordable or by offering “good deals.” However, the findings from the current study did not provide support for this, as participants’ self-reported use of betting inducements was not found to be a significant predictor of problem gambling scores in the multivariate analysis.

Although we were unable to find a clear positive association between exposure to sports betting marketing and problem gambling, it does not rule out the role that sports betting marketing has to play in contributing to young people’s gambling issues. Participants who reported that they were more likely to bet after encountering sports betting advertising exhibited higher scores for problem gambling. These findings are consistent with those of Kristiansen and Severin-Nielsen (2021), who also observed a relationship between self-perceived advertising impact and problematic gambling, but in a younger sample. This suggests that sports betting advertising effectively acts as a stimulus or trigger for individuals with gambling problems to engage in gambling activities. Similarly, young people who perceived themselves as more vulnerable to betting inducements, meaning they were more inclined to engage in riskier gambling behaviours when betting with inducements, were more likely to have high problem gambling scores. For non-problem or low-risk bettors, inducements could potentially enhance the enjoyment or affordability of betting. However, for those struggling with gambling, they can serve to intensify their betting behaviours, increasing the excitement associated with sports betting and resulting in a lack of control even when faced with negative consequences (Hing et al., 2014, 2018; Lopez-Gonzalez et al., 2019).

The results of this study emphasise a significant contributing factor in the development of problematic gambling behaviours among young people: their perceived vulnerability to advertising and incentives. This finding supports Binde’s (2008) assertion that problem gamblers may be particularly sensitive to gambling advertising. Based on our findings, we propose that policymakers prioritise efforts to reduce the salience of sports betting marketing in general while also focusing specifically on reducing the promotion of betting inducements to mitigate sports betting-related harm among young people.

### Limitations and Suggestions for Future Research

The current study found that individuals who exhibit negative urgency are more likely to engage in problem gambling. This suggests that those high in negative urgency turn to gambling as a means of escaping negative emotions, subsequently leading to the development of gambling-related problems. Future studies might consider incorporating a measure to assess the occurrence of negative emotional states among participants. It is likely that those who habitually use gambling to cope during periods of emotional distress and concurrently experience these distressing states frequently are at a heightened risk for developing gambling problems.

Since the current study was cross-sectional in nature, we cannot infer directionality, and how these risk factors develop over time or whether they are predictive of long-term problem gambling. Consequently, it is crucial to interpret these results only as they pertain to young people. Although the study found that exposure to sports betting advertising was not predictive of problem gambling, it may play a role in introducing people to sports betting, which may serve as a catalyst for later problem gambling. Therefore, further longitudinal research is needed to investigate the causal relationship between early exposure to betting marketing and the subsequent development of problem gambling later in life.

## Conclusion

These findings suggest that sports betting marketing is predictive of problem gambling scores when controlling for demographic, psychological, and behavioural predictors. However, the impact of marketing is primarily attributed to people’s perceived vulnerability to its influence. Therefore, it is likely that betting advertising plays a role in triggering gambling-related urges, and inducements play a role in intensifying gambling behaviours among young problem gamblers. Consequently, further regulations on betting advertising may be an effective measure for reducing harm among young problem gamblers.
